# Child Marriage Acceptability Index (CMAI) as an essential indicator: an investigation in South and Central Sulawesi, Indonesia

**DOI:** 10.1186/s41256-022-00252-4

**Published:** 2022-09-26

**Authors:** Muliani Ratnaningsih, Heribertus Rinto Wibowo, Nicholas J. Goodwin, Ade Ayu Kartika Sari Rezki, R. Ridwan, Ratnakanya Nitya Hadyani, Emilie Minnick, Derry Fahrizal Ulum, Tanti Kosmiyati Kostaman, Sitti Nur Faizah

**Affiliations:** 1 PT Tulodo Indonesia Makmur, 18 Office Park, Lt. 22, Suite E, F, G TB Simatupang Street Kav 18, South Jakarta, 12520 Indonesia; 2 UNICEF Indonesia, World Trade Center II, 22nd Floor, Kav. 31, Jend. Sudirman Street No.8, RT.8/RW.3, Kuningan, Karet Kuningan, South Jakarta, 12920 Indonesia; 3 Sekolah Tinggi Ilmu Kesehatan Tamalatea Makassar, Makassar, Indonesia

**Keywords:** Child Marriage Acceptability Index (CMAI), Dowry, Sexual and gender-based violence (SGBV)

## Abstract

**Background:**

Child marriage remains an important social issue in Indonesia. Child marriages were reported from 14.67% in 2008 to 10.82% in 2019. However, 22 out of 34 provinces in Indonesia still had high child marriage rates above the national average in 2019. This study aims to assess child marriage acceptability in the two locations in Indonesia by gender inequality, financial security, education rates, legal frameworks, dowry, and sexual and gender-based violence (SGBV).

**Methods:**

This study used a quantitative approach with a cross-sectional study design. A total of 1000 respondents consisting of 500 households in Bone District, South Sulawesi and 500 households in Palu, Sigi, and Donggala District in Central Sulawesi participated in the study. Data analyses were conducted based on the Child Marriage Acceptability Index (CMAI) using the bivariate correlation, ANOVA (analysis of variance), and logistic regression.

**Results:**

This study found several significant factors that contributed to child marriage acceptance in Central and South Sulawesi: household financial security (p = 0.016), dowry (p < 0.001) and legal frameworks (p = 0.017) based on ANOVA analysis. After conducting a bivariate correlation, dowry (p < 0.001) and sexual and gender-based violence (p < 0.001) remain significant factors. Dowry (*p* < 0.001), with expected B = 0.122, and sexual and gender-based violence (p < 0.001, with expected B = 0.064) remains significant after the linear regression analysis.

**Conclusions:**

Dowry practice and sexual and gender-based violence were the most significant factors contributing to child marriage acceptance in Central and South Sulawesi. There is a need to conduct interventions to prevent child marriage, including providing sexual and reproductive health education.

**Supplementary Information:**

The online version contains supplementary material available at 10.1186/s41256-022-00252-4.

## Introduction

Child marriage remains an important social issue worldwide, and it is a violation of human rights. Girls are more vulnerable to child marriage compared to boys [1]. UNICEF reported that there had been a decline in the global prevalence of child marriage in the last decade [2]. However, there are still around 12 million girls who experience child marriage per year. The highest prevalence of child marriage is in sub-Saharan Africa and South Asia, where an estimated 38% and 30% of girls marry before 18 years, respectively [3]. Countries and societies with high gender inequality (e.g., laws and customs that exclude girls from decision-making or economic and political rights) are more likely to feature a high prevalence of child marriage [4].

Child marriage remains an important social issue in Indonesia. UNICEF data ranks Indonesia eighth in the world and second highest in Southeast Asia in terms of the percentage of marriages in which at least one of the spouses is under 18 years [5]. A decrease in child marriages was reported in Indonesia—from 14.67% in 2008 to 10.82% in 2019. However, 22 out of 34 provinces in Indonesia still had high child marriage rates above the national average in 2019. South Kalimantan had the highest rate (21.2%) followed by Central Kalimantan (20.2%) and West Sulawesi (19.2%). On the other hand, the Indonesian government has a target to decrease the child marriage prevalence to 8.74% in 2024 as part of its national development strategy [6].

Several studies have explored the contributing factors to child marriage in Indonesia which include socio-economic factors such as poverty, cultural norms, and low educational opportunities [7–9]. Those living in poverty were more likely to marry early. In many cases, parents marry off their children to lessen their economic burden [10]. Those dropping out of school were also more likely to marry early [11]. Unwanted pregnancy also played a significant contributing factor to child marriage. Child marriage is assumed to be a way to prevent risky sexual behaviour between girls and boys (to avoid sin or *zinah*) [12]. UNICEF also reported that those living in rural areas were more likely to marry early compared to those in urban areas [6].

The focus areas of the study were Bone District in South Sulawesi, and Palu, Sigi and Donggala District in Central Sulawesi. Based on the Statistics Bureau Agency, the total population in Bone in 2020 was 801,775 people consisting of 391,682 men and 410,093 women. The total population in Palu was 373,218 people consisting of 187,389 men and 185,829 women in 2020. The total population in Sigi was 257,585 people consisting of Donggala was 300,436 people. There has been a decrease in child marriage cases in Central Sulawesi since 2016. However, the child marriage rate in Central Sulawesi (15.8%) was still higher than the national average (11.2%) in 2018, which remained high after the earthquake and tsunami disaster in the same year. In 2019, the prevalence of child marriage in Central Sulawesi was 16.3% (an increase of 0.5% compared to 2018), still higher than the national rate (10.82%). The child marriage prevalence in South Sulawesi in 2018 was 14.1%, higher than the national level (11.2%), and in 2019, the prevalence was 12.1%, still higher than the national rate (10.82%) [13].

In several countries, families practice dowry, the transfer of assets and goods from the bride to the groom’s family at the time of marriage, or practice bridewealth, payment by the groom or his family to the bride’s parents [13]. In Indonesia, the bride price is called *Mas Kawin* or *Mahar* which is compulsory for marriage in Islamise. *Mahr* is a mandatory gift from a prospective husband to his future wife. The Presidential Instruction of Indonesia No.1 1991 concerning The Dissemination of the Compilation of the Islamic Law stated that *mahr* can be in the form of goods, money, or services that are not against the Islamic Law (article 1) and the groom has an obligation to pay *mahr* to the bride with agreement from both parties [14]. There are several types of *Mahr*: money, jewellery, animals or religion-related assets such as Al-Quran and other worship equipment. *Mahr* in etymology means dowry. It was stated in the *Surah An-Nisa* In the Al-Quran (chapter four): *“Dan berikanlah maskawin (mahar) kepada perempuan (yang kamu nikahi) sebagai pemberian yang penuh kerelaan* (“Give a dowry to a woman (whom you marry) as a gift full of willingness). Men have an obligation to pay *Mas Kawin* or *mahr* and it is a compulsory bride price [13]. The bride price practice may be different in each area in Indonesia including its amount. It is influenced by the prospective bride’s social status, education level and occupation.

Many *Bugis* people also live in South and Central Sulawesi. In the socio-cultural contexts of *Bugis adat,* both women and men of Bone play significant (and different) roles in their families and society. Although Bugis culture follows a patriarchal line, women are critically involved in both domestic and public matters. Women are consulted by men, appear in public, mediate their husbands’ social relationships, make business decisions and even play a political leadership role [14, 15]. The female *arumponé* (royal leaders)—ruling the society as a kingdom for centuries—is clear historical evidence of these practices [15]. Among low-income families, the roles of women are even more significant in adapting to the day-to-day precarity and informal nature of poor people's lives. Bone district has the highest number of women as the head of the family in South Sulawesi (26%), with 6% of women as the primary breadwinners. Only 23.5% of them had identity cards (*Kartu Tanda Penduduk*/KTP), while 29% of them could not read or write [17].

Gender-based violence is also a contributing factor to child marriage and has a negative impact on child marriage. A study in 34 countries found that women who married as children were more likely to report physical and/or sexual violence from their parents compared with those who married as adults [14]. In 2019, at the Nairobi Summit on the International Conference on Population and Development (ICPD) 25, Indonesia committed to implement strategic policies and campaigns and increase the national and sub-national budgets to end gender-based violence and harmful practices, and revise the Marriage Law 1974, by increasing the age of first marriage to at least 19 years for both boys and girls [15]. Previously, according to Marriage Law No. 1/1974, the legal age for girls in Indonesia to get married with parental consent is 16 years old. However, the Marriage Law was amended in September 2019 to Law No. 16/2019, increasing the legal age for girls to get married to 19 years old, the same age as boys [17].

There are many factors that contribute to child marriage such as poverty, cultural norms, low educational opportunities, prospective bride's social status, education level, and occupation. All of these factors are calculated by the Child Marriage Acceptability Index (CMAI). The CMAI is designed to ‘score’ communities according to the presence/absence of indicators associated with norms and attitudes about when a girl’s marriage is necessary, desirable, acceptable or unacceptable or an index for measuring environmental factors associated with the acceptability of child marriage. It is important to measure the index as it could assess levels of ‘risk’ of child marriage acceptability in a community (anticipate how likely a community is to be accepting of the practice of child marriage) and could help to design interventions that target these ‘drivers’ more effectively and track their impact. The CMAI could track and measure changes in both structural drivers associated with child marriage acceptability, and levels of (predicted) community acceptability over time. The CMAI can be useful for two of these two locations, as the child marriage rate was still high. Thus, this study will explore child marriage acceptability in these two locations in Indonesia by measuring several drivers such as gender inequality, financial security, education rates, legal frameworks, dowry, and sexual and gender-based violence (SGBV).

## Methods

### Research design

The study used a cross-sectional design, with researchers making observations of variables at certain times. The participants consisted of parents in households with children aged 13–15 years in two locations, Central Sulawesi, and South Sulawesi. The minimum number of participants was calculated based on Hulley et al. (2013) (p. 57) method, where N (sample) = 16: (standardized size effect)^2^. The standardized size effect to be used is 0.3, with a confidence interval of 95%, and power (β) of 80%. From the number of samples that have been calculated, researchers add an additional sample up to 10% of the total including adding samples in the control area. So that the number of samples becomes 500 for each location. The total sample that participated in this study was 1000 respondents (500 respondents from Bone, South Sulawesi and 500 respondents from Palu, Sigi, Donggala, and Central Sulawesi).

This research has received ethical approval from the Ethics Commission for Research and Community Health Service, Faculty of Public Health, University of Indonesia (No. 256/UN2.F10/PPM.00.02/2019) from the Provincial Government of South and Central Sulawesi. Respondents in this study signed an informed consent which contained information about the length of the interview, the confidentiality of the data provided, voluntary participation of the respondents, no potential hazards generated after the research, and the research results would not be used by anyone other than researchers and interested parties.

### Questionnaire development

The CMAI is comprised of a set of indicators (and associated variables) that explain the presence of environmental factors associated with the acceptability of child marriage. The main indicators for the CMAI included household financial security, education level, legal framework, dowry and sexual and gender-based violence. This study used 21 questions on attitudes and perceptions that relate to various elements of acceptability of child marriage based on the CMAI questionnaire developed by Plan International and ACMI. Each question has its own score, for example, the ideal age for marriage, disparity in ideal age for marriage for girls compared to boys, and so on. For the perception of child marriage, the responses were arranged along a 7-level Likert scale consisting of: strongly disagree, disagree, slightly disagree, neither disagree nor agree, slightly agree, agree and strongly agree. For testing of each question from CMAI, there are two types of statistical tests used: the validity test uses the Corrected item-total Correlation, while the reliability test uses Cronbach's Alpha (0.693). The questionnaires were developed based on the Child Marriage Acceptability Index (CMAI) developed by Asia Child Marriage Initiative (ACMI) from Plan International (Additional file 1).

The purpose of using this indicator will make it easier to calculate each indicator in the CMAI. Financial security indicator, the household income was categorized into six groups: those whose income is in the top 10% of the national average; 4th quintile (top 60–80%), 3rd quintile (40–60%), 2nd quintile (bottom 20–40%) and 1st quintile (bottom 20%) and those living below poverty line). It also measured whether households had sufficient food, clothes, medicines, and school items; then the responses were classified into always, most of the time, sometimes and never. The education indicator was measured by classifying head of household education into having formal/mainstream education, basic education/madrassa, and no education). The legal framework was measured by asking whether households have corrected knowledge of the marriage law including whether they register their marriage and own identity documents. The dowry variable was measured by exploring whether marriage involves dowry practices and whether younger brides or grooms require a lower dowry price. The last indicator, sexual and gender-based violence (SGBV) measures the acceptance of sexual violence against women/girls and male control over marriage, and the belief that child marriage prevents sexual harassment (Additional file 2).

### Data collection

Respondents aged above 30 years and over from 8 villages in Bone, South Sulawesi and 19 villages in Palu, Sigi, and Donggala in Central Sulawesi who have children aged 13–15 years were recruited. This study was conducted to provide a situation analysis, particularly on the knowledge, attitudes, and perceptions among households to develop recommendations for the child marriage prevention program in these areas. The child marriage prevention campaign (BERANI) targets adolescents in secondary school (aged 13–15 years) and parents of children aged 13–15 years. Thus, we used the same age groups reference from the program to conduct the study. So, it will be in line with the program. With the help of the village leaders, we mapped the houses and determined which respondents were visited by using random techniques, for example with the distance of every 10 houses, and if there were no families with children aged 13–15 years, we visited the house next door. The data collection used the mWater Surveyor App on tablets and smart handphones. The survey took approximately 50–60 min to complete. The data that has been collected is stored by Tulodo Indonesia. To access the full data set of this CMAI research, please contact UNICEF Indonesia and/or the corresponding author.

### Data processing and analysis

To analyse the quantitative data, the SPSS 25.0 for PC was used. Descriptive statistics were used to analyse demographic data, such as sex, age, income, and education status. To answer the research question, inferential statistics were used. For testing each question from CMAI, there are two types of statistical tests used: the validity test uses the Corrected item-total Correlation, while the reliability test uses Cronbach's Alpha (0.693) (Additional file 1).

For the household financial security indicator, the household income was categorized into six groups: those whose income is in the top 10% of the national average; 4th quintile (top 60–80%), 3rd quintile (40–60%), 2nd quintile (bottom 20–40%) and 1st quintile (bottom 20%) and those living below poverty line). It also measured whether households had sufficient food, clothes, medicines, and school items; then the responses were classified into always, most of the time, sometimes, and never. The education indicator was measured by classifying the head of households’ education into having formal/mainstream education, basic education/madrassa, and no education). The legal framework was measured by asking whether households have corrected knowledge of the marriage law including whether they register their marriage and own identity documents. The dowry variable was measured by exploring whether marriage involves dowry practices and whether younger brides or grooms require a lower dowry price. The last indicator, sexual and gender-based violence (SGBV) measures the acceptance of sexual violence against women/girls and male control over marriage, and the belief that child marriage prevents sexual harassment. After calculating the acceptability scores of each variable, a series of statistical tests, including bivariate correlation, ANOVA and regression analysis were conducted to test the associations between the independent variable and the child marriage acceptability scores.

## Results

### Demographic characteristics of the study respondents

A total of 1000 respondents of parents with children aged 13–15 years participated in the survey, 50.0% from South Sulawesi and 50% from Central Sulawesi. Most of the respondents were female (83.2%) whilst 16.8% were male. Most of the respondents had income between IDR 500,000 and 1,000,000 (53.2%) whilst 20.2% had income less than IDR 500,000; income between IDR 2,000,000 and 3,000,000 (13.0%), income between 1,000,001 and 2,000,000 (6.0%) (Table 1).

**Table 1:** Sample characteristics (n = 1000)

Sample characteristics	%
*Area*	
South Sulawesi	50.0
Central Sulawesi	50.0
*Sex*	
Female	83.2
Male	16.8
*Income (IDR)*	
Less than 500,000	20.2
500,000–1,000,000	53.2
1,000,001–2,000,000	6.0
2,000,001–3,000,000	13.0
3,000,001–4,000,000	4.4
4,000,001–5,000,000	1.3
5,000,001–6,000,000	0.4
6,000,001–7,000,000	0.6
Above 7,000,000	0.9

For education, the index is divided into two questions: father's education and mother's education. For father’s education, 33.4% had completed elementary school, 23.6% graduated from senior high school, and 19.6% completed junior high school. For mothers’ education, 31.5% completed elementary school, 23.4% graduated from high school, and 23.3% graduated from junior high school (Table 2).

**Table 2:** Education of family member (n = 1000)

Education of family member	%
*Education of father*	
No school/out of school	2.7
Graduated from Islamic Elementary School	0.1
Graduated from Islamic Junior High School	1.3
Graduated from Islamic Senior High School	1.1
Graduated from Elementary School	33.4
Graduated from Junior High School	19.6
Graduated from Senior High School	23.6
Not complete Islamic Elementary School	0.2
Not complete Islamic Junior High School	0.1
Not complete Islamic Senior High School	0.3
Not complete Elementary School	7.2
Not complete Junior High School	2.5
Not complete Senior High School	2.1
University or College	5.8
*Education of mother*	
No school/out of school	1.8
Graduated from Islamic Elementary School	0.1
Graduated from Islamic Junior High School	2.1
Graduated from Islamic Senior High School	0.7
Graduated from Elementary School	31.5
Graduated from Junior High School	23.3
Graduated from Senior High School	23.4
Not complete Islamic Elementary School	0.2
Not complete Islamic Junior High School	0.2
Not complete Islamic Senior High School	0.1
Not complete Elementary School	5.3
Not complete Junior High School	2.5
Not complete Senior High School	1.8
University or College	7.0

### Child Marriage Acceptability Index: ideal age for marriage

In total, there are twenty questions comprising the Child Marriage Acceptability Index (CMAI). Table 3 showed the ideal age of marriage for girls, the disparity in the ideal age of marriage for girls compared to boys, lowest and highest acceptable age of marriage for girls. Most respondents said ideal marriage was between 19 and 20 years (31.1%), whilst a high number reported 23–25 years (28.7%) and 21–22 years (26.6%). Most respondents said the disparity in the ideal age of marriage for girls compared to boys was 3 years (37.0%), then 2 years (20.1%) and 5 years (26.9%). Most respondents said the lowest acceptable age of marriage for girls was 18 years or more (64.7%), whilst 17 years (20.6%) and 15 years (5.4%). Most respondents said the highest acceptable age of marriage for girls was 30–40 years (39.5%), then 25–29 years (27.3%) and above 40 years (11.2%).

**Table 3:** Frequency table of ideal age for marriage (n = 1000)

Ideal age for marriage	%
*Ideal age of marriage of girls*	
> 25 years	7.4
23–25 years	28.7
21–22 years	26.6
19–20 years	31.1
18 years	4.6
17 years	1.1
≤ 17 years	0.5
*Disparity in ideal age of marriage for girls compared to boys*	
0 year	3.9
1 year	6.5
2 years	20.1
3 years	37.0
4 years	11.6
5 years	26.9
> 5 years	4.0
*Lowest acceptable age of marriage for girls*	
18 years or more	64.7
17 years	20.6
16 years	4.7
15 years	5.4
14 years	2.1
13 years	2.2
12 years or below	0.3
*Highest acceptable age of marriage for girls*	
No upper limit	7.4
> 40 years	11.2
30–40 years	39.5
25–29 years	27.3
21–24 years	11.1
19–20 years	3.5
18 years or lower	0

### Perceptions around child marriage

The Child Marriage Acceptability Index (CMAI) also explored the perception of household members around child marriage using sixteen statements. A total of 76.0% of respondents disagreed with the statement that a girl was ready for marriage once she started menstruating whilst 19.7% agreed. A total of 81.1% of respondents disagreed with the statement that there are advantages to the marriage of girls under 18 years whilst 14.6% agreed. A total of 80.9% agreed with the statement that there are disadvantages for girls getting married under 18 years whilst 15.6% disagreed. A total of 69.2% disagreed with the statement that marrying girls can help protect family honours/reputation whilst 26.2% agreed. A total of 71.8% disagreed with the statement that girls who give birth between 15 and 18 years are more likely to have a healthy pregnancy/baby whilst 17.6% agreed. A total of 78.2% disagreed with the statement that marrying young girls can help resolve financial problems in the family whilst 17.7% agreed. A total of 72.5% disagreed with the statement that marrying young girls can help provide them security whilst 23.3% agreed. A total of 67.7% disagreed with the statement that early marriage of girls can help prevent sexual violence, assault, and harassment whilst 26.9% agreed. A total of 60.4% disagreed with the statement that early marriage of boys can help prevent sexual violence, assault, and harassment whilst 26.8% agreed (Table 4).

**Table 4:** Perceptions on child marriage acceptability (n = 1000)

Statements	STA	A	SLA	N	SLD	D	STD
A girl is ready for marriage once she starts menstruating	1.0	15.3	3.4	4.3	6.5	61.2	8.3
There are advantages to the marriage of girls under 18 years	0.9	9.1	4.6	4.3	7.2	65.8	8.1
There are disadvantages for girls getting married under 18 years	16.3	59.8	4.8	3.5	5.0	8.8	1.8
Marrying girls can help protect family honour/reputation	1.2	17.4	7.6	4.6	11.1	52.6	5.5
Girls who give birth between 15 and 18 years are more likely to have a healthy pregnancy/baby	1.0	11.9	4.7	10.6	11.5	52.1	8.2
Marrying girl young can help resolve financial problems in the family	1.3	10.2	6.2	4.1	10.1	61.0	7.1
Marrying young girls can help provide them security	1.0	13.8	8.5	4.2	12.1	55.1	5.3
Early marriage of girls can help prevent sexual violence, assault, and harassment	0.9	15.7	10.3	5.4	12.9	49.5	5.3
Early marriage of boys can help prevent sexual violence, assault, and harassment	1.3	16.7	8.8	5.3	4.8	50.8	4.8
Marrying under 18 years is likely to have a negative impact on a girl education	22.4	61.2	3.2	2.8	3.3	7.0	0.1
Marrying a young girl is preferable because younger brides are more obedient and respectful of their husbands	1.7	14.2	7.5	8.5	13.0	50.7	4.4
Even if a girl does not want to be married, she should honour the decisions/wishes of her family	4.2	21.7	7.7	5.6	10.1	46.2	4.5
Younger brides require a lower dowry than older brides	1.6	10.8	5.1	13.6	13.3	52.0	3.6
A girl should never be forced or compelled into marriage	27.2	57.1	2.7	3.0	3.1	5.6	1.3
It is sometimes okay to beat or punish a girl when he dishonours her family	1.8	19.5	8.8	3.7	10.2	48.7	7.3
A wife should be subservient to her husband	31.9	59.5	1.7	2.1	2.5	1.9	0.4
Men should be the heads of their household	35.8	56.4	1.5	2.5	1.8	1.5	0.5

*STA:* strongly agree, *A:* agree, *SLA:* slightly agree, *N:* neither disagree nor agree (neutral), *SLD:* slightly disagree, *D:* disagree, *STD:* strongly disagree

A total of 86.8% agreed with the statement that marrying under 18 years is likely to have a negative impact on a girl's education whilst disagreed (10.4%). A total of 68.1% disagreed with the statement that marrying a young girl is preferable because younger brides are more obedient and respectful of their husbands whilst 23.4% agreed. A total of 60.8% disagreed with the statement that even if a girl does not want to be married, she should honour the decisions/wishes of her family whilst 33.6% agreed. A total of 68.9% disagreed with the statement younger brides require a lower dowry than older brides whilst 17.5% agreed. A total of 87.1% agreed with the statement that a girl should never be forced or compelled into marriage whilst 10.0% disagreed. A total of 66.2% disagreed with the statement that it is sometimes okay to beat or punish a girl when she dishonours her family whilst 30.1% agreed. A total of 93.1% agreed with the statement that a wife should be subservient to her husband whilst 4.8% disagreed. A total of 93.7% agreed with the statement that men should be the heads of their household whilst 3.8% disagreed.

### Analysis of variance: Child Marriage Acceptable Index

Table 5 shows the results of ANOVA analysis for the following variables: gender, household financial security, education, legal framework, dowry, sexual-based violence, and gender which were statistically significant when tested with the CMAI indicators. ANOVA statistical tests showed results that household financial security (p = 0.016, F = 1.126), legal frameworks (p = 0.017, F = 1.421), and dowry (p = 0.000, F = 1.189) were significant as contributing factors to child marriage using the CMAI. The statistical test results showed that financial security, legal frameworks, and dowry were the contributing factors to child marriage in South and Central Sulawesi.

**Table 5:** Analysis of variance (ANOVA)

Variable	SS	dF	MS	F	Sig
*Gender*				0.470	1.000
Between groups	116.034	68	1.706		
Within groups	33,378.366	931	3.629		
Household financial security*				1.427	0.016*
Between groups	740.665	68	10.892		
Within groups	7106.734	931	7.633		
*Education*				1.126	0.232
Between groups	164.133	68	2.414		
Within groups	1995.291	931	2.143		
Legal frameworks*				1.421	0.017*
Between groups	3306.610	68	48.627		
Within groups	31,859.790	931	34.221		
Dowry*				2.514	0.000*
Between groups	1083.830	68	15.939		
Within groups	5901.926	931	6.339		
*Sexual and gender-based violence*				1.289	0.063
Between groups	6567.493	68	96.581		
Within groups	76,342.256	931	74.946		

*SS:* sum of squares, *dF:* degree of freedom, *MS:* mean square

*Significant. If the value of Sig (p < 0.005) and value F count > F table (F count > 0.729)

### Correlation Variables on Child Marriage Acceptable Index

Furthermore, Table 6 used bivariate correlation tests (Pearson Correlation) statistical tests with the same variables. Pearson correlation statistical tests showed results that dowry and SGBV (sexual and gender-based violence) significantly have an impact on the CMAI indicators. The bivariate correlation (Pearson Correlation) statistical tests showed results that dowry (p < 0.000, r = 0.216) and SGBV (p < 0.000, r = 0.111) were significant as contributing factors with the CMAI. The statistical test results showed that dowry and sexual and gender-based violence (SGBV) was the contributing factors to child marriage in South and Central Sulawesi using the CMAI.

**Table 6:** Bivariate correlation (Pearson correlation)

Variable	Pearson correlation (r)	Sig. (2-tailed)
Gender	0.005	0.879
Household financial security	− 0.056	0.079
Education	− 0.036	0.262
Legal frameworks	0.032	0.312
Dowry*	0.216	0.000*
Sexual and gender-based violence*	0.111	0.000*

^*^Significant. If the value of Sig p < 0.005 and value r count > r table (r count > 0.062)

From the statistical test results in Tables 5 and 6, variables that are statistically significant or p value < 0.005 will be entered into the linear regression analysis. Table 7 shows the results of statistical tests using linear regression analysis which show the most significant variables with the CMAI index. The regression linear statistical analysis showed that dowry (p = 0.000, B = 0.209) and SGBV (sexual and gender-based violence (p = 0.001, B = 0.101) remained significant contributing factors to the CMAI.

**Table 7:** Regression linear

Variable	Standardized coefficients beta (B)	t	Sig	95% confidence interval	
				Lower limit	Upper limit
Gender	0.000	0.006	0.995	− 0.012	0.012
Household financial security	− 0.054	− 1.725	0.085	− 0.047	0.003
Education	− 0.028	− 0.892	0.373	− 0.047	0.003
Legal frameworks	0.028	0.893	0.372	− 0.013	0.035
Dowry*	0.209	6.763	0.000*	0.086	0.157
Sexual and gender-based violence*	0.101	3.254	0.001*	0.025	0.100

^*^Significant. If value of Sig (p < 0.05) and value t count > t two-side (t count > 1.96)

## Discussion

### Dowry practices and child marriage

This study found that dowry practices have significant contributing factors to child marriage in Indonesia, particularly in Central and South Sulawesi. Several studies showed that financial transactions around marriage may also contribute to the practice of child marriage, especially in contexts of poverty and vulnerability [16, 18–21].

In communities where the groom or his family pays a bride price at the time of marriage, which is often the case in parts of Africa, parents may benefit from marrying their daughters early if waiting increases bride prices [18]. By contrast, in communities where the bride brings resources at the time of marriage (dowry, which is more prevalent in South Asia), the required dowry to be paid by parents may be lower if the bride is younger [19]. Marrying a daughter at a younger age also reduces the investments that a family must make in her education, without necessarily curtailing future returns to those investments if those returns benefit mostly the groom’s family. This may lead parents to reap immediate benefits from an early marriage even if this is not in the long-term interest of the girl marrying early [20].

Nonetheless, as a result of the mounting pressure to control girls' behaviour and marry them early, the practise of dowry is widespread and demands are increasing, a view supported by participants in this research. As one older participant in research notes: “*When I got married, there was no thought of the groom getting a single dime. Rather the groom had to pay 100 Bangladesh taka (USD1.18) to bring the wife along with him. And that money had to be paid to the father of the bride. The groom had to pay the cost of the ceremony. Now, it's the other way around. If you go to any girl or the girl's father, they ask for money or things. (Older man, Gaibandha)”* and “*Now it is quite impossible to marry without a dowry. The amount is 10,000 taka (USD 118). 20,000 taka (USD 236) and 30.000 takas (USD 354) and more… now it is quite impossible. Most are not married without a dowry. There is a great demand [on the family for] money, though they forget to know the name of the girl but not forget about dowry. It is very funny, the cow that is given as dowry, firstly they wanted to see it. Their curiosity is about how nice the cow is. It is like a market of dowry, they take [marry] a little girl but do not take her without a dowry. (Older man Gaibandha)*” [20].

In India where dowry payments are common, shocks may reduce the probability of child marriage, possibly, because a girl’s or a boy's family is unable to meet the dowry requirements. Cultural norms also heavily influenced child marriage. In societies where bride price payment is practised (i.e., the groom’s family provides assets to the bride’s family in exchange for marriage), the bride’s family may reap immediate financial benefits from marrying their daughter [21]. In other contexts, a younger bride is more desirable as she has more time to commit to her new family and bear more children. Thus, where a bride's price is paid, the bride’s family may gain greater benefit the younger their daughter is, which may motivate parents to marry their daughter early. Similarly, in circumstances where dowry payment is practised, a smaller dowry may be required for a younger bride, so parents may be incentivised to marry their daughter at a young age to avoid the increasing cost [22].

In Bugis culture, the most dominant ethnic in South Sulawesi and some in Central Sulawesi, there is a form of a bridewealth practice named *uang panai*. A study from South Sulawesi reported that bridewealth practice (*uang panai*) was determined by the women's social status, age, education level, and pride of the bride and her family. *Uang panai* is paid by the groom to the bride and her family to conduct the wedding ceremony [23]. As *uang panai* has social value in Bugis culture, it may explain our findings that bridewealth/dowry practices were widely accepted by the community.

A study in Donggala in Central Sulawesi province reported that virginity was also considered the main factor in determining the amount of dowry besides the level of education, employment status, religion, behaviour and beauty. It can be measured whether the girls are already pregnant (*kawin kecelakaan*) or still virgins (*kawin adat*). Those that are *kawin adat* will have a higher dowry amount compared to those who experienced *kawin kecelakaan* (*kawin lari*, getting pregnant, and marriage because of being caught red-handed by a traditional leader) [24].

The amount of money and dowry brought by the prospective groom is determined by the results of the family deliberations of the prospective husband and wife. The study shows the level of socio-economic status of the woman's family whom she intends to marry also greatly determines the size of the marriage fee. This status is usually measured by virginity, level of education, employment status, religion, behaviour and beauty. A community leader in *Tanjung Batu* stated the following: *“If a woman who wants to get married has high social status (the woman is still a virgin, works as a civil servant, bachelor, religion, physical appearance), then the dowry for this is at least 30 million rupiahs (USD 2100). But if they just graduated from high school…., only 10–20 million (USD700-1400).”* [25].

### The link between household financial security and child marriage

This study also found that financial security including income also contributed to the acceptability of child marriage in Central and South Sulawesi. The high prevalence of child marriage in Indonesia is related to poverty. A study in Indonesia reported that child marriage especially for girls has a tendency to limit their income generation capacity so they can have a higher standard of living. In this study, women’s economic conditions by age group were classified into five categories: lowest 20%, lowest 20–40%, lowest 40–60%, lowest 60–80%, and highest 20%. The lowest 20% and lowest 20–40% welfare status reflect poor economic conditions. Using logistic regression, it was found that there is a negative correlation between child marriage and income per capita [25]. The findings support our study as there was also a negative correlation between household financial security or income with child marriage acceptability.

Several studies also have explored that poverty and the lack of viable income-generating options for girls and young women are important factors contributing to high child marriage rates. Skills and financial incentives are sometimes linked to investing in education in girls and/or on the condition that they do not marry until the age of 18 [26]. Child marriage leads women to have children earlier and more children over their lifetime than if they had married later. It affects girls’ educational attainment and literacy negatively, thereby curtailing future opportunities for them to compete for well-paying jobs [18].

A lack of their own income and financial planning skills has the potential to reduce bargaining power for women within the household as well as investment in their children, affecting future generations and contributing to girls getting married at an early age [27]. Similarly, early marriage is also likely to limit the earning capacity of women by reducing their education, work experience before marriage, and ability to work outside the home while married. Even if women who experience child marriage, later contribute more to household income, the improvement in income alone may not always ensure good health outcomes for them. They and their family members (e.g., their babies) will still be more likely to experience stunting, physical disabilities, and risk of degenerative diseases in the future [28, 29].

Child marriage is often considered a way to lessen the economic burden on the family. Supporting household economic security may provide an acceptable alternative to marriage and increase the value and contribution of the children, particularly girls to her family, for example, having a higher income. The intervention can be conducted by providing scholarships or support for children to access education and conducting livelihood programs.

### Knowledge of legal framework to prevent child marriage

This study found that the knowledge of the legal framework is a significant contributing factor to the acceptability of child marriage in Central and South Sulawesi by using ANOVA, even though it did not remain significant after the regression linear analysis. The legal framework was measured by asking whether households must correct knowledge of the marriage law including whether they register their marriage and own identity documents.

Knowledge of the legal framework of marriage in Indonesia may contribute to child marriage prevention. Indonesia has made progress by increasing the legal minimum of marriage for girls. Previously, according to Marriage Law No. 1/1974, Indonesia allowed girls aged 16 years and below to marry [30]. The Marriage Law was amended in September 2019, and it increased the age that girls can be married from 16 to 19 years, the same age as boys. The legal framework is an enabling environment factor for child marriage prevention, even though some studies reported that it is not sufficient to create lasting change [31].

Field data from West Bengal reveal that the PCMA (types of legal law codes in India) had success in reducing the number of such marriages or punishing its practitioners. Thus, the state had only six registered police cases of child marriage in 2008. Data for the year 2009 are not yet available. The district of Malda did not register a single case of child marriage until September 2010. The district has state-imposed an injunction to prohibit child marriage in recent times. This reveals that the new law has motivated the relevant persons to implement the law. Conversely, the existing DSWOs and CMPOs (types of legal aid agencies in India) need to wait for changes in social attitudes to enable them to initiate any real action against the violators of the law [21].

Knowledge of the legal framework to prevent child marriage influences the decision of a family to marry off their child or not. However, the implementation of the legal framework on marriage prevention in different countries is different because of the factors in the CMAI that may support child marriage occurring in the family. To our findings in Indonesia, there are legal products produced to prevent child marriage, but due to the causative factor, namely unwanted pregnancy in adolescents, the practice of child marriage occurs in Indonesia. In contrast to India, this country has almost the same population as Indonesia, but the application of the law to prevent child marriage is strong enough to suppress child marriage.

### Interconnectivity of sexual and gender-based violence and child marriage

This study found that SGBV (sexual and gender-based violence) was a significant contributing factor to the acceptability of child marriage in Central and South Sulawesi using ANOVA analysis and linear regression analysis. The sexual and gender-based violence (SGBV) measure explored the acceptance of sexual violence against women/girls and male control over marriage as well as the belief that child marriage prevents sexual harassment.

Child marriage is a manifestation of gender-based violence and a violation of the fundamental human rights of women as many girls are forced into marriage against their free will and consent. It is harmful to children as it robs them of their childhood innocence and turns them into “adults” prematurely. For girls, the age at which they are married renders them unable to negotiate safe sex and are therefore vulnerable to sexually transmitted diseases and domestic violence. The child brides are forced into sexual intercourse with their spouses as soon as they are married resulting in very early pregnancy and resulting in vesical vaginal fistula (VVF) obstetric fistula when such girls give birth eventually at very tender ages [32].

In societies where gender norms devalue girls’ positions, girls are more likely to experience violence within marriages. They are also more likely to experience physical and sexual abuse than those who marry later. Girls not only experience abuse from their husbands but also from other family members. As girls enter marriage, they may be forced to have marital sex earlier than they are ready. Furthermore, it may lead to adolescent pregnancy with a high risk of complications during birth and a negative impact on the young mother and the baby. Gender-based violence can also harm children’s mental and physical health and increase the perception of violence as acceptable. Children who witness violence are also more likely to perpetrate violence as adults. Various studies reveal that there was a strong likelihood that violent, or child abuse will become a continuing cycle of violence. The rates of abuse are higher among women whose husbands were abused as children or who saw their mothers being abused [33].

Gender-based-power relations between young wives and husbands, parents and in-laws are not equal. The unequal gender-based power relations in female child marriage practices in poor families are related to the limitation of knowledge and reproduction of power [34]. Unequal gender relations are continuously reproduced through the imposition of negative social labelling on girls. The prevention of female child marriage requires a comprehensive approach by addressing the social and cultural values, especially promoting equal gender relations. One of the solutions is empowerment based on an equal gender perspective [35]. Several recommendations to prevent child marriage and increase youth’s knowledge about the impact of child marriage and the possibility of getting pregnant out of wedlock if they engage in risky sexual behaviour.

The limitation of this study used the cross-sectional study design, it assessed the independent and dependent simultaneously (capturing one single time). The limitation of the study is that we cannot draw the causal relationship between the independent and dependent variables. The association between the variables are predictive in nature as we measured the respondents’ knowledge, attitudes, and perceptions toward child marriage issues. Further study will be needed to analyse the relationship between these two variables, for example, to analyse the determinant of child marriage by focusing more on the sample from populations who experienced child marriage.

## Conclusions

Overall, this study concluded that based on CMAI, there were some respondents that have positive perceptions toward child marriage. This study also found that in areas with dowry practices, child marriage is more likely to be accepted by society as the amount of dowry is also determined by factors such as age, education level, girls’ status, and so on. Dowry practices will continue as it is part of the culture in South Sulawesi, however, there is a need to change the community's perception of dowry practices in order to address child marriage and gender inequality. For example, there is a need to change the community's perceptions and practices around lower *uang panai* among the younger girls and change the norms that girls below 19 years should not be married. Sexual and gender-based violence was also a significant contributing factor to the acceptability of child marriage. In societies where girls are not valued the same as boys, violence and abuse are more likely. On the other hand, SGBV also has a negative impact on child marriage. Women who were married as children were more likely to experience violence and abuse compared to those married as adults.

To protect children’s rights, particularly girls, there is a need to combat child marriage by developing interventions and policies to prevent children from marrying and protect those who do marry early from violence and abuse. There is also a need to ensure that all women have the resources and support to leave abusive relationships. Sexual and reproductive health education is also needed for parents and adolescents to increase their knowledge of the issues and improve their behaviours. As Indonesia has increased the minimum age of marriage for women, it could support the child marriage prevention program at the national and sub-national levels. Indonesia has made a progress in preventing child marriage as in 2019, Indonesia had amended the Marriage Law and raised the age that girls can be married with parental permission from 16 to 19 years, in line with the age for boys. Indonesia has also developed a national strategy for child marriage prevention. As one of the protective factors against child marriage is education, there is a need to strengthen the collaboration of across government sectors and other relevant stakeholders to reaffirm the 12-year compulsory education law in Indonesia. As more children participated in the compulsory education program, it will help to end marriages below 18 years.

## Supplementary Information

**Additional file 1.** Coding and calculation of the child marriage acceptability index used in the study.

**Additional file 2.** Validity and reliability test result of Child Marriage Acceptability Index questions.

## Abbreviations

ANOVA: Analysis of variance
BERANI: Better Reproductive Health and Rights for All in Indonesia
UNICEF: United Nations International Children's Emergency Fund
CMAI: Child Marriage Acceptance Index
CMPO: Calcutta Metropolitan Planning Organisation
DSWO: District Social Welfare Office
ICPD: International Conference on Population and Development
IDR: Indonesia Rupiah (Indonesia currency)
KTP: *Kartu Tanda Penduduk* (Indonesia Identity Card)
PCMA: Prohibition of Child Marriage Act
SGBV: Sexual and gender-based violence
STA: Strongly Agree
A: Agree
SLA: Slightly Agree
N: Neither disagree nor agree (neutral)
SLD: Slightly Disagree
D: Disagree
STD: Strongly Disagree
USD: United States Dollar
VVF: Vesical vaginal fistula
