# Supplementary material for: Child Marriage Acceptability Index (CMAI) as an essential indicator: an investigation in South and Central Sulawesi, Indonesia
Source: Glob Health Res Policy. 2022 Sep 26;7:32. doi: 10.1186/s41256-022-00252-4 (PMC9511735; doi:10.1186/s41256-022-00252-4)
Supplement: Supplementary file 1 — Additional file 1. Coding and calculation of the child marriage acceptability index used in the study. [file 41256_2022_252_MOESM1_ESM.docx]

**Annex 1.** Validity and reliability test of Child Marriage Acceptability Index questions

| CMAI questions | Validity test | | Reliability test |
| --- | --- | --- | --- |
|  | Pearson Correlation | Sig | Cronbach's Alpha if Item Deleted |
| Ideal age of marriage for girls* | 0.277 | 0.000 | 0.688 |
| Disparity in ideal age of marriage for  girls compared to boys* | 0.083 | 0.009 | 0.697 |
| Lowest acceptable age of marriage for  girls* | 0.346 | 0.000 | 0.685 |
| Highest acceptable age of marriage for  girls* | 0.162 | 0.000 | 0.692 |
| A girl is ready for marriage once she starts menstruating* | 0.526 | 0.000 | 0.673 |
| There are advantages to marriage of girls under 18 years* | 0.589 | 0.000 | 0.672 |
| There are disadvantages for girls getting married under 18 years* | 0.072 | 0.044 | 0.702 |
| Marrying girls can help protect family honour/reputation* | 0.609 | 0.000 | 0.668 |
| Girls who give birth between 15-18 years are more likely to have a healthy pregnancy/baby* | 0.520 | 0.000 | 0.675 |
| Marrying girl young can help resolve financial problems in the family* | 0.626 | 0.000 | 0.669 |
| Marrying young girls can help provide them security* | 0.659 | 0.000 | 0.666 |
| Early marriage of girls can help prevent sexual violence, assault, and harassment* | 0.657 | 0.000 | 0.665 |
| Early marriage of boys can help prevent sexual violence, assault, and harassment* | 0.655 | 0.000 | 0.665 |
| Marrying under 18 years is likely to have a negative impact on a girl's education* | 0.361 | 0.000 | 0.683 |
| Marrying a young girl is preferable because younger brides are more obedient and respectful of their husbands* | 0.359 | 0.000 | 0.682 |
| Even if a girl does not want to be married, she should honour the decisions/wishes of her family* | 0.254 | 0.000 | 0.688 |
| Younger brides require a lower dowry than older brides* | 0.185 | 0.000 | 0.693 |
| A girl should never be forced or compelled into marriage* | 0.135 | 0.000 | 0.693 |
| It is sometimes okay to beat or punish a girl when he dishonours her family* | 0.127 | 0.000 | 0.693 |
| A wife should be subservient to her husband. | 0.167 | 0.000 | 0.692 |
| Men should be the heads of their household* | 0.140 | 0.000 | 0.693 |

**Valid and reliable. If the value of Sig <0.005 and value r count > r table (r count > 0.062) in Pearson Correlation and Cronbach's Alpha test.*
