# Supplementary material for: Child Marriage Acceptability Index (CMAI) as an essential indicator: an investigation in South and Central Sulawesi, Indonesia
Source: Glob Health Res Policy. 2022 Sep 26;7:32. doi: 10.1186/s41256-022-00252-4 (PMC9511735; doi:10.1186/s41256-022-00252-4)
Supplement: Supplementary file 2 — Additional file 2. Validity and reliability test result of Child Marriage Acceptability Index questions. [file 41256_2022_252_MOESM2_ESM.pdf]

Child Marriage Acceptability Index (CMAI) - South & Central Sulawesi

Variabel	Dimension		Category			
Gender	**Gender**		5 = women/female (high risk)	0 = man/male (low risk)		
Household financial security	**Average monthly income**		**Area**	Bone<br>Rp 2,860,382	Pasigala<br>Rp 2,123,040	Sulawesi<br>Rp 2,491,711
			(Income is in top 10% of national average) = 0	Rp 2,860,382	Rp 2,123,040	Rp 2,491,711
			(4th quintile/ top 80%) = 1	Rp 2,288,306	Rp 1,698,432	Rp 1,993,369
			(4th quintile/ top 60%) = 1	Rp 1,716,229	Rp 1,273,824	Rp 1,495,027
			(Middle/3rd quintile/60%) = 2	Rp 1,716,229	Rp 1,273,824	Rp 1,495,027
			(Middle/3rd quintile/ 40%) = 2	Rp 1,144,153	Rp 849,216	Rp 996,684
			(2nd quintile/ bottom 40%) = 3	Rp 1,144,153	Rp 849,216	Rp 996,684
			(2nd quintile/ bottom 20%) = 3	Rp 572,076	Rp 424,608	Rp 498,342
			(1st quintile bottom 20%) = 4	Rp 572,076	Rp 424,608	Rp 498,342
			(Living below the poverty line) = 5	below Rp 572,076	below Rp 434,608	below Rp 498,342
	**How often does the household have enough of the following items (all)**	**Sufficient food so as not to go hungry**	Never = 4 Rarely = 3 Sometimes = 2 Often = 1 Always = 0			
		**Regular Medicines**	Never = 4 Rarely = 3 Sometimes = 2 Often = 1 Always = 0			
		**School items (uniforms/ books etc.**	Never = 4 Rarely = 3 Sometimes = 2 Often = 1 Always = 0			
Education	**Caregiver's/Mother's Education (all)**		Formal/mainstream education) = 0<br>(Basic education/ Madrassa) = 3<br>(Lower than basic education/No education) = 5			
	**Father's Education (all)**		Formal/ mainstream education) = 0<br>(Basic education/ Madrassa) = 3<br>(Lower than basic education/No education) = 5			
Legal Frameworks	**Correct knowledge of (existing) law which prohibits child marriage (all)**		(There is no law) = 10<br>(unable to correctly identify law) = 10<br>(Able to identify an existing, protective law) = 0			
	**Did the participant register their marriage? (married respondents only)**		(No) = 10<br>(Yes) = 0			
	**Does the participant possess and identity document? (all)**		(No) = 10<br>(Yes) = 0			

Variabel	Dimension	Category
Dowry	**Do marriages in the community involve the exchange/ agreement regarding dowry? (all)**	(No) = 0<br>(Yes) = 10
	**Do younger brides require a lower dowry price? (Views of men/boys)**	(Strongly agree) = 10<br>(Agree) = 8<br>(Neutral) = 5<br>(Disagree) = 2<br>(Strongly Disagree) = 0 (Not applicable/ no dowry) = 0
	**Do younger brides require a lower dowry price? (Views of women/girls)**	(Strongly agree) = 10<br>(Agree) = 8<br>(Neutral) = 5<br>(Disagree) = 2<br>(Strongly Disagree) = 0 (Not applicable/ no dowry) = 0
SGBV (Sexual and Gender Based Violence)	**Acceptance of sexual violence against women/ girls and male dominance/ control within marriage (all)**	(Strongly agree) = 10<br>(Agree) = 8<br>(Neutral) = 5<br>(Disagree) = 2<br>(Strongly Disagree) = 0
	**Cultural/social belief that early marriage of girls prevents sexual harassment/ violence (views of men/boys)**	(Strongly agree) = 10<br>(Agree) = 8<br>(Neutral) = 5<br>(Disagree) = 2<br>(Strongly Disagree) = 0
	**Cultural/social belief that early marriage of girls prevents sexual harassment/ violence (views of men/boys)**	(Strongly agree) = 10<br>(Agree) = 8<br>(Neutral) = 5<br>(Disagree) = 2<br>(Strongly Disagree) = 0
Child Marriage Acceptability Index (CMAI)	**Ideal age of marriage for girls.**	(1) = >25; (2) = 23-25; (3) = 21-22; (4) = 19-20; (5) = 18; (6) = 16-17; (7) = <16
	**Disparity in ideal age of marriage for girls compared to boys.**	(1) = 0 years; (2) = 1 year; (3) = 2 years; (4) = 3 years; (5) = 4 years; (6) = 5 years; (7) = <5 years
	**Lowest acceptable age of marriage for girls.**	(1) = 18 years or more; (2) = 17; (3) = 16; (4) = 15; (5) = 14; (6) = 13; (7) = 12 years or below
	**Highest acceptable age of marriage for girls.**	(1) = no upper limit; (2) = >40; (3) = 30-40; (4) = 25-29; (5) = 21-24; (6) = 19-20; (7) = 18 years or lower
	**A girl is ready for marriage once she starts menstruating**	(1) = strongly disagree; (2) = disagree; (3) = slightly disagree; (4) = neutral; (5) = slightly agree; (6) = agree; (7) = strongly agree.

Variabel	Dimension	Category
	**There are advantages to marriage of girls under 18 years**	(1) = strongly disagree; (2) = disagree; (3) = slightly disagree; (4) = neutral; (5) = slightly agree; (6) = agree; (7) = strongly agree.
	**There are disadvantages for girls getting married under 18 years.**	(1) = strongly disagree; (2) = disagree; (3) = slightly disagree; (4) = neutral; (5) = slightly agree; (6) = agree; (7) = strongly agree.
	**Marrying girls young can help protect family honour/ reputation.**	(1) = strongly disagree; (2) = disagree; (3) = slightly disagree; (4) = neutral; (5) = slightly agree; (6) = agree; (7) = strongly agree.
	**Girls who give birth between 15-18 years are more likely to have a healthy pregnancy/ baby (compared to girls over 18).**	(1) = strongly disagree; (2) = disagree; (3) = slightly disagree; (4) = neutral; (5) = slightly agree; (6) = agree; (7) = strongly agree.
	**Marrying girls young can help resolve financial problems in the family.**	(1) = strongly disagree; (2) = disagree; (3) = slightly disagree; (4) = neutral; (5) = slightly agree; (6) = agree; (7) = strongly agree.
	**Marrying girls young can help provide them security.**	(1) = strongly disagree; (2) = disagree; (3) = slightly disagree; (4) = neutral; (5) = slightly agree; (6) = agree; (7) = strongly agree.
	**Early marriage of girls can help prevent sexual violence, assault and harassment.**	(1) = strongly disagree; (2) = disagree; (3) = slightly disagree; (4) = neutral; (5) = slightly agree; (6) = agree; (7) = strongly agree.
	**Early marriage of boys can help prevent perpetration of sexual violence, assault and harassment.**	(1) = strongly disagree; (2) = disagree; (3) = slightly disagree; (4) = neutral; (5) = slightly agree; (6) = agree; (7) = strongly agree.
	**Marrying under 18 years is likely to have a negative impact on a girls' education.**	(1) = strongly agree; (2) = agree; (3) = slightly agree; (4) = neutral; (5) = slightly disagree; (6) = disagree; (7) = strongly disagree.
	**Marrying a girl young is preferable because younger brides are more obedient and respectful of their husbands.**	(1) = strongly disagree; (2) = disagree; (3) = slightly disagree; (4) = neutral; (5) = slightly agree; (6) = agree; (7) = strongly agree.
	**Even if a girl does not want to be married, she should honour the decisions/ wishes of her family.**	(1) = strongly disagree; (2) = disagree; (3) = slightly disagree; (4) = neutral; (5) = slightly agree; (6) = agree; (7) = strongly agree.

Variabel	Dimension	Category
	**Younger brides require a lower dowry than older brides.**	(1) = strongly disagree; (2) = disagree; (3) = slightly disagree; (4) = neutral; (5) = slightly agree; (6) = agree; (7) = strongly agree.
	**A girl should never be forced or compelled into marriage.**	(1) = strongly agree; (2) = agree; (3) = slightly agree; (4) = neutral; (5) = slightly disagree; (6) = disagree; (7) = strongly disagree.
	**It is sometimes ok to beat or punish a girl when she dishonours her family.**	(1) = strongly disagree; (2) = disagree; (3) = slightly disagree; (4) = neutral; (5) = slightly agree; (6) = agree; (7) = strongly agree.
	**A wife should be subservient to her husband.**	(1) = strongly disagree; (2) = disagree; (3) = slightly disagree; (4) = neutral; (5) = slightly agree; (6) = agree; (7) = strongly agree.
	**Men should be the heads of their household.**	(1) = strongly disagree; (2) = disagree; (3) = slightly disagree; (4) = neutral; (5) = slightly agree; (6) = agree; (7) = strongly agree.
